# Cost-effectiveness of sacituzumab govitecan versus chemotherapy in patients with relapsed or refractory metastatic triple-negative breast cancer

**DOI:** 10.1186/s12913-023-09728-6

**Published:** 2023-06-29

**Authors:** Jiao Xie, SiNi Li, YaMin Li, JianHe Li

**Affiliations:** 1 Department of Otolaryngology-Head and Neck Surgery, The Second Xiangya Hospital, Central South University, Changsha, 410011 Hunan China; 2 Clinical Nursing Teaching and Research Section, The Second Xiangya Hospital, Central South University, Changsha, 410011 China; 3 The Nethersole School of Nursing, Faculty of Medicine, The Chinese University of Hong Kong, Shatin, Hong Kong; 4 Present Address: The Second Xiangya Hospital, Central South University, 139 Renmin Middle Road, Changsha, 410011 Hunan China; 5 Department of Pharmacy, The Second Xiangya Hospital, Central South University, Changsha, 410011 China

**Keywords:** Cost-effectiveness, Microsimulation model, Triple-negative breast cancer, Sacituzumab Govitecan, Chemotherapy

## Abstract

**Background:**

The effectiveness of sacituzumab govitecan for metastatic triple-negative breast cancer (TNBC) has been reported in recent research, however, the value of the effectiveness and cost of sacituzumab govitecan is still unclear.

**Methods:**

A microsimulation model was developed using data from the ASCENT trial to assess the cost-effectiveness of sacituzumab govitecan for patients with relapsed or refractory metastatic TNBC over a lifetime. Model inputs, including clinical data, patient characteristics, and direct medical costs, were based on the ASCENT trial, public databases, and published literature. The primary outcomes of the model were the incremental cost-effectiveness ratio (ICER) and quality-adjusted life-years (QALYs). Univariate and probabilistic sensitivity analysis (PSA) and multiple scenario analyses were performed to address the uncertainty of the model.

**Results:**

Our results revealed that sacituzumab govitecan versus chemotherapy costs $293,037 and yielded an additional 0.2340 of QALYs in the whole population with metastatic TNBC, leading to an ICER of $1,252,295 gained. And in the population with metastatic TNBC without brain metastasis, the sacituzumab govitecan versus chemotherapy costs $309,949 and obtained an extra 0.2633 of QALYs, which resulted in an ICER of $1,177,171/QALYs. Univariate analyses indicated that the model outcomes were most sensitive to the drug cost of sacituzumab govitecan, the utility of progression-free disease, and the utility of progressed disease.

**Conclusion:**

From the US payer perspective, sacituzumab govitecan is unlikely to be a cost-effective option for patients with relapsed or refractory metastatic TNBC compared with chemotherapy. Based on the value standpoint, a price decrease of sacituzumab govitecan is expected to increase the cost-effectiveness of sacituzumab govitecan in patients with metastatic TNBC.

**Supplementary Information:**

The online version contains supplementary material available at 10.1186/s12913-023-09728-6.

## Introduction

Breast cancer (BC) is the most common type of female tumor and has the second highest mortality rate in the United States, with over 276,480 cases diagnosed and 42,170 deaths occurring in 2020 [1]. Triple-negative breast cancer (TNBC), a phenotypic subtype of BC that is defined as negative for hormone receptors and human epidermal growth factor receptor 2 (HER2), accounts for approximately 10–20% of all BC patients and is a highly aggressive disease with a poor diagnosis and outcomes (the 5-year survival rate is less than 30% for patients with advanced TNBC) [2–4]. Moreover, the management of TNBC accounts for 5–10% of all cancer expenditures and for 0.5% of the total health care budget in the United States (US) [5]. Therefore, the financial burden of TNBC on patients has been gradually increasing globally and has become an issue that should be seriously taken into account [6].

Although international guidelines recommend the use of single-agent chemotherapy as the primary systemic treatment for metastatic TNBC, chemotherapy is related to poor response rates and short progression-free survival (PFS) [7–12]. Therefore, the potential value of novel regimens for TNBC treatment needs to be determined. Sacituzumab govitecan, as the first antibody–drug conjugate targeting anti-trophoblast cell surface antigen 2 (Trop-2) and selectively delivering SN-38, was approved by the Food and Drug Administration (FDA) in 2020 [13]. Recently, the ASCENT, an open-label phase 3 randomized controlled trial (RCT), reported the efficacy and safety of sacituzumab govitecan compared with single-agent chemotherapy of the physician’s choice (vinorelbine, capecitabine, gemcitabine or eribulin) in patients with relapsed or refractory metastatic TNBC [14]. The results revealed that sacituzumab govitecan notably prolonged the median PFS and overall survival (OS) compared with chemotherapy ([5.6 months versus 1.7 months; hazard ratio (HR) for progression or death, 0.41; 95% confidence interval [CI], 0.32–0.52; *P* < 0.001] and [12.1 months versus 1.7 months; HR for death, 0.48; 95% CI, 0.38 to 0.59; *P*< 0.001], respectively) [14]. In addition, due to considering the high prevalence of brain metastasis in TNBC (ranged from 25 to 46%) [15], which may lead to different disease and financial burdens to patients with TNBC, the ASCENT trial also reported the efficacy of sacituzumab govitecan versus chemotherapy in TNBC patients without brain metastasis [14]. And similar to the full trial population of patients with TNBC, the clinical benefit of sacituzumab govitecan versus chemotherapy in PFS (5.6 months versus 1.7 months; HR for progression or death, 0.41; 95% CI, 0.32–0.52; *P* < 0.001) and OS (12.1 months versus 6.7 months; HR for death, 0.48; 95% CI, 0.38 to 0.59; *P*< 0.001) were identified in TNBC patients without brain metastasis [14]. For the full population, treatment-related grade 3 or higher adverse events (AEs) were more often reported in the sacituzumab govitecan group than in the chemotherapy group (24.8% versus 21.0%) [14]. Consequently, sacituzumab govitecan is likely to be an attractive option to treat patients with relapsed or refractory metastatic TNBC. It is also crucial for both clinicians and decision makers to consider the value of agents when making healthcare decisions to optimally allocate limited healthcare resources [16]. Therefore, the objective of this study was to investigate the cost-effectiveness of sacituzumab govitecan versus chemotherapy for metastatic TNBC from the US payer perspective.

## Material and methods

### Analytics overview

**Fig. 1:**
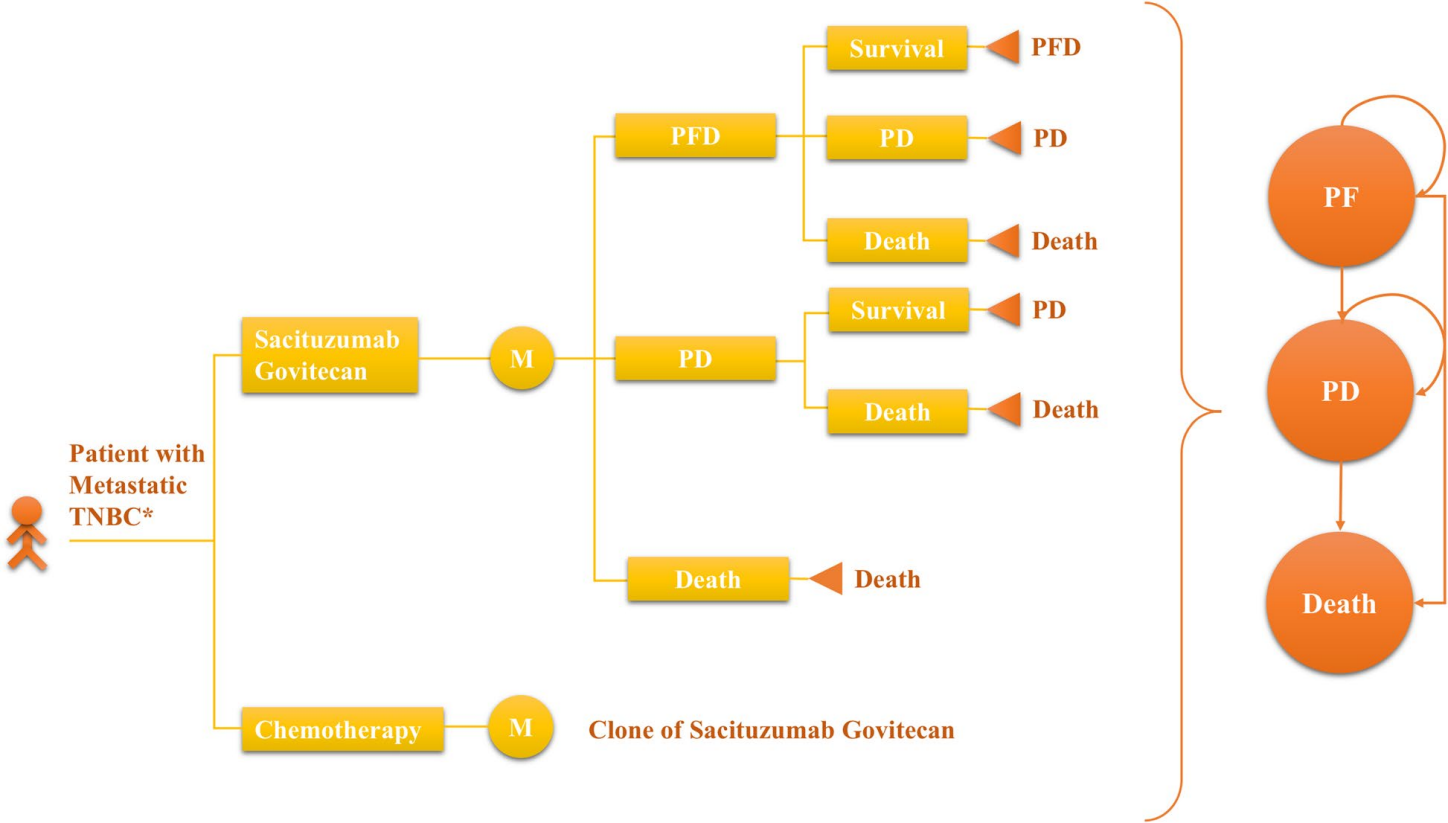
Model Structure. *Patients included full population or patients without brain metastatic; PFD = progression-free disease; PD = progressed disease

A decision-analytic model (microsimulation) was constructed to compare the lifetime clinical and economic outcomes of sacituzumab govitecan with those of chemotherapy for metastatic TNBC by using TreeAge Pro (TreeAge Software, Williamstown, MA) (Fig. 1). The decision model included two scenarios: the whole population (scenario 1) and patients without brain metastasis (scenario 2). In both scenarios, patients received one of two interventions: sacituzumab govitecan or single-agent chemotherapy of the physician’s choice (54% eribulin, 20% vinorelbine, 13% capecitabine, or 12% gemcitabine). After the disease progressed, we assumed that patients would receive best supportive care (BSC). The transition model in this study included the following three mutually exclusive health states to specifically reflect the disease course of metastatic TNBC: progression-free disease (PFD), progressed disease (PD), and death (Fig. 1) [16]. All of the simulated patients began their path through the model in the PF health stage, and depending on the transition probability, they may either progress to PD (PF → PD) or the death state. And patients who have experienced PD may remain in their present health state or progress to death (PD → Death). The model cycle length was 21 days (keeping with the treatment schedule reported in the ASCENT trial [14]), and the time horizon (10 years) was used to estimate the health outcomes, including total costs, quality-adjusted life-years (QALYs), life-years (LY), and incremental cost-effectiveness ratios (ICERs). A half-cycle correction was applied in the model. The baseline patient characteristics were obtained to mirror the respective RCT (ASCENT trial) [14] (eTable 1 in the Supplement). During each model cycle, the hypothetical patients were transitioned among the three health states according to transition probabilities that were derived from the ASCENT trial [14].

### Clinical data inputs

The transition probabilities for sacituzumab govitecan and chemotherapy were estimated by the survival curves of the ASCENT trial (at least trial follow-up), and patients switched among different health states on the basis of transition probabilities. Moreover, we extrapolated over the model time horizon using the standard extrapolation technique described by Guyot et al. [17] In summary, the data points of PFS and OS obtained from Kaplan–Meier curves were extracted by using GetData Graph Digitizer software (http://www.getdata-graph-digitizer.com/) to generate pseudoindividual patient-level data. Using parametric survival functions fitted to trial survival data (PFS and OS), the distribution of patients among three health states were calculated. And those reconstructed survival data were used to fit the following 6 parametric survival functions: exponential, generalized gamma, Weibull, Gompertz, log-normal, and log-logistic. Based on visual inspection and the goodness-of-fit (Akaike information criterion) method, the appropriate distribution was chosen for all PFS and OS curves to the observed data and clinical plausibility of long-term extrapolations. The PFS and OS plots developed by using the pseudoindividual level-patient data and the lifetime-predicted curves by using parametric survival models are illustrated in eFigures 1–4 in the Supplement. And the results showed that the Weibull model provided the greatest statistical fit (lowest AIC) to the OS for sacituzumab govitecan in the full TNBC population and in the TNBC patients without brain metastasis. Moreover, loglogistic distribution was used to predict PFS for sacituzumab govitecan, OS, and PFS for chemotherapy in the full TNBC population and in TNBC patients without brain metastasis since it showed good statistical fit and visual plausibility. Therefore, the formula 1 (loglogistic distribution) was used to estimate the transition probabilities for disease progression (PF → PD) in sacituzumab govitecan and chemotherapy arms [18]. Moreover, the formula 2 (Weibull distribution) was used to assess the transition probabilities for death from progression-free disease state (PF → death) or the death from post-progression (PD → death) [18]. However, we also incorporated the age-specified background mortality rate in the model using the 2019 US Life Table [19]. Therefore, the maximum value was selected between the probability for death state and background mortality. The formulae 3 was applied to transform the rates in the life table to the transition probability [18]. The results of the key clinical parameters of transition probabilities were listed in the Table 1.

**Table 1:** Model parameters: baseline values, ranges, and distributions for sensitivity analysis

		Range			
Variable	Baseline value	Minimum	Maximum	Distribution	Reference
Survival model of full population					
Sacituzumab Govitecan					
OS	γ = 1.4281			Weibull	Estimated
	λ = 15.6779				
PFS	γ = 1.739			Loglogistic	Estimated
	λ = 4.675				
Chemotherapy					
OS	γ = 1.933			Loglogistic	Estimated
	λ = 6.636				
PFS	γ = 2.251			Loglogistic	Estimated
	λ = 2.348				
Survival model of no brain metastases					
Sacituzumab Govitecan					
OS	γ = 1.445			Weibull	Estimated
	λ = 16.598				
PFS	γ = 1.799			Loglogistic	Estimated
	λ = 5.152				
Chemotherapy					
OS	γ = 1.87			Loglogistic	Estimated
	λ = 6.759				
PFS	γ = 2.552			Loglogistic	Estimated
	λ = 2.266				
Drug costs, $					
Sacituzumab govitecan, per 2.5 mg	29.505	23.60	35.41	Gamma	20
Eribulin per 0.1 mg	117.762	94.21	141.31	Gamma	20
Vinorelbine, per 10 mg	9.544	7.64	11.45	Gamma	20
Capecitabine, per 150 mg	0.745	0.60	0.89	Gamma	20
Gemcitabine, per 200 mg	3.968	3.17	4.76	Gamma	20
Other costs input, $					
Administration cost, IV infusion, single or initial drug	148.3	118.64	177.93	Gamma	25
CT scan per cycle	114.47	91.57	137.36	Gamma	2
Supportive care per month	4614	3461	5768	Gamma	28
Terminal care	9574	7180	11,967	Gamma	27
Follow-up per month	1146	842	1450	Gamma	3
Management of SAEs	16,016	5001	18,397	Gamma	26
Quality-of-life (utility)					
PFD	0.85	0.64	1	Beta	16
PD	0.52	0.39	0.65	Beta	16
Disutility due to Grade 3–4 AEs	0.28	0.21	0.35	Beta	16
Discount rate (%)	3	0	5	Uniform	21

*OS* Overall survival, *PFS* Progression-free survival, *AEs* Adverse events

1 $$P({t}_{u})=1-\left\{\{\frac{1}{1+{\left(\frac{t-u}{\uplambda }\right)}^{\upgamma }}\}/\{\frac{1}{1+{\left(\frac{t}{\uplambda }\right)}^{\upgamma }}\}\right\}$$

2 $$\mathrm{P}({t}_{u})=1-\mathrm{exp}\{{\uplambda (\mathrm{t}-\mathrm{u})}^{\upgamma }-{\mathrm{\lambda t}}^{\upgamma }\}$$

3 $$\mathrm{P}=1-\mathrm{exp }(-\mathrm{rt})$$

*The P represents the transition probability, and t is the time; t_u_ represents that t is now evaluated as integer multiples of the cycle length of the model, u; γ is the shape parameter; λ is the scale parameter; and r is the rate.

### Cost and utility inputs

All the costs and utilities incorporated in the model are presented in Table 1. We considered only direct medical costs, including the cost of drug acquisition, administration, computed tomography (CT), management of AEs, BSC, follow-up and end-of-life care, and reported them in 2021 USD (Table 1). The US consumer price index was used to calculate the costs inflated to 2021 values [20], and costs and utilities were discounted by an annual rate of 3% [21].

Based on the ASCENT trial, sacituzumab govitecan at a dose of 10 mg/kg of body weight was intravenously administered on days 1 and 8 of a 21-day cycle, while single-agent chemotherapy was administered as follows: eribulin at a dose of 1.4 mg/m^2^ of the body surface area intravenously on days 1 and 8 of a 21-day cycle; vinorelbine at a dose of 25 mg/m^2^ intravenously on day 1 weekly; capecitabine at a dose of 1000 to 1250 mg/m^2^ (the mean dose 1125 mg was used in the model) orally twice daily on days 1 to 14 of a 21-day cycle; and gemcitabine at a dose of 800 to 1200 mg/m^2^(the mean dose 1000 mg was used in the model) intravenously on days 1, 8, and 15 of a 28-day cycle. The unit drug prices were estimated on the basis of the 2021 average sale price from the Centers for Medicare & Medicaid Services (CMS) [22]. The body surface area (1.79 m^2^) and patient weight (70 kg) were used to calculate the drug cost per cycle [23, 24]. The cost of administration was obtained from the 2021 CMS Physician Fee Schedule [25]. We incorporated only AEs of at least grade 3, and the overall costs associated with those AEs were obtained from a real-world study [26]. The overall costs related to CT, BSC, follow-up and end-of-life care were derived from previous reports [2, 3, 27, 28].

The utility scores for PFS and PD, which ranged from 0 (death) to 1 (perfect health), were collected from previously published studies [16]. In this study, the PFD and PD states were assigned utility values of 0.85 and 0.52, respectively. Disutility values associated with AEs (-0.28), obtained from Wu et al., were also incorporated in the model, and we assumed that AEs were incurred only in the first cycle. [16, 29]

### Sensitivity analyses

To evaluate the robustness of the model and test the uncertainty of the model related to variables, a series of sensitivity analyses, including univariate sensitivity analysis, probability sensitivity analysis (PSA) and scenario analysis, were conducted. In the univariate sensitivity analysis, critical input parameters were changed successively to their respective lower and upper limitations, which were derived from their 95% CIs or by adjusting by a variance of 20% from the base-case values to determine the influence of the ICER, in accordance with the existing cost-effectiveness analysis approach [30–32]. The Cholesky decomposition matrix method was employed in the PSA to randomly extracted correlating variables from multivariate normal distributions to estimate the uncertainty of correlating survival parameters (e.g., scale and shape parameters). The variance–covariance matrix, Cholesky decomposition matrix, and Cholesky equation with the random normal distribution for all survival parameters were listed in Supplementary eTable 5.

For the PSA, a Monte Carlo simulation of 5000 iterations of 5000 patients was performed by using a specific pattern of distributions to sample the key parameters (Table 1), and a cost-effectiveness acceptability curve (CEAC) was developed to illustrate the likelihood that sacituzumab govitecan could be regarded as a cost-effective option at different willingness-to-pay (WTP) levels for health gains (QALYs). The current WTP threshold in the US is $150,000/QALYs [33]; therefore, sacituzumab govitecan could be considered a very cost-effective treatment if the ICER of sacituzumab govitecan vs chemotherapy falls below this WTP threshold.

We analyzed four scenarios in the whole population in this study. In the first scenario, we changed the patient age at which treatment was started to assess the influence of the ICER in the model. In the second scenario, the time horizon was varied to 1, 3, and 5 years to evaluate the impact of PFS and OS extrapolations used in the model. In the third scenario, we assumed that only 80% or 50% of patients would receive BSC after disease progression to simulate that certain patients would discontinue treatment due to other causes in clinical practice. Finally, we reduced the price of sacituzumab govitecan to 80%, 50%, and 20% from its original cost.

## Results

### Base-case analysis

Fifty thousand patients were simulated for the two treatments to decrease the effect of statistical fluctuations on the cost and health outcomes, and the results are listed in Table 2. In baseline scenario 1 (i.e., the sacituzumab govitecan strategy in the whole population), the mean cost and QALYs were $395,470 and 0.7297, respectively, while those of chemotherapy were $102,433 and 0.4957, respectively. For LY, sacituzumab govitecan provided 1.1373 LY, which was 0.3175 LY more than chemotherapy provided. The sacituzumab govitecan arm was required to pay an additional $293,037, resulting in an ICER of $922,951/LY or $1,252,295/QALYs compared with the chemotherapy arm (Table 2). In baseline scenario 2 (i.e., the sacituzumab govitecan strategy in patients without brain metastasis), the mean cost, QALYs, and LY were $418,402, 0.7779, and 1.1971, respectively, while those of chemotherapy were $108,453, 0.5146, and 0.8531, respectively. The ICER of sacituzumab govitecan vs. chemotherapy was $901,015/LY or $1,177,171/QALYs among patients without brain metastasis.

**Table 2:** Summary base case results

Arm	Total LYs	Total QALYs	Total Costs	Inc. LYs	Inc. QALYs	Inc. Costs	ICER/LY	ICER/QALY
Full population (SD; 95% CI)								
Chemotherapy	0.8198 (0.9842; 95% CI [0.8111, 0.8284])	0.4957 (0.5213; 95% CI [0.4912, 0.5003])	102,433 (102,433; 95% CI [101311, 103555])	-	-	-	-	-
Sacituzumab govitecan	1.1373 (0.7915; 95% CI [1.1303, 1.1442])	0.7297 (0.4776; 95% CI [0.7255, 0.7339])	395,470 (294,420; 95% CI [392889, 398051])	0.3175 (0.9577; 95%CI [0.3091, 0.3259])	0.2340 (0.50; 95% CI [0.2296, 0.2383])	293,037 (269,428; 95% CI [290676, 295399])	922,951	1,252,295
Patients without BM (SD; 95% CI)								
Chemotherapy	0.8531 (1.0454; 95% CI [0.8439, 0.8623])	0.5146 (0.5762, 95% CI [0.5095, 0.5196])	108,453 (142,119; 95% CI [107208, 109699])	-	-	-	-	-
Sacituzumab govitecan	1.1971 (0.8255; 95% CI [1.1899, 1.2043])	0.7779 (0.5026, 95% CI [0.7735, 0.7823])	418,402 (310,158; 95% CI [415683, 421121])	0.3440 (1.0356; 95% CI [0.3349, 0.3531])	0.2633 (0.5563; 95% CI [0.2584, 0.2682])	309,949 (289,320; 95% CI [307413, 312485])	901,015	1,177,171

### One-way sensitivity and probability analyses

**Fig. 2:**
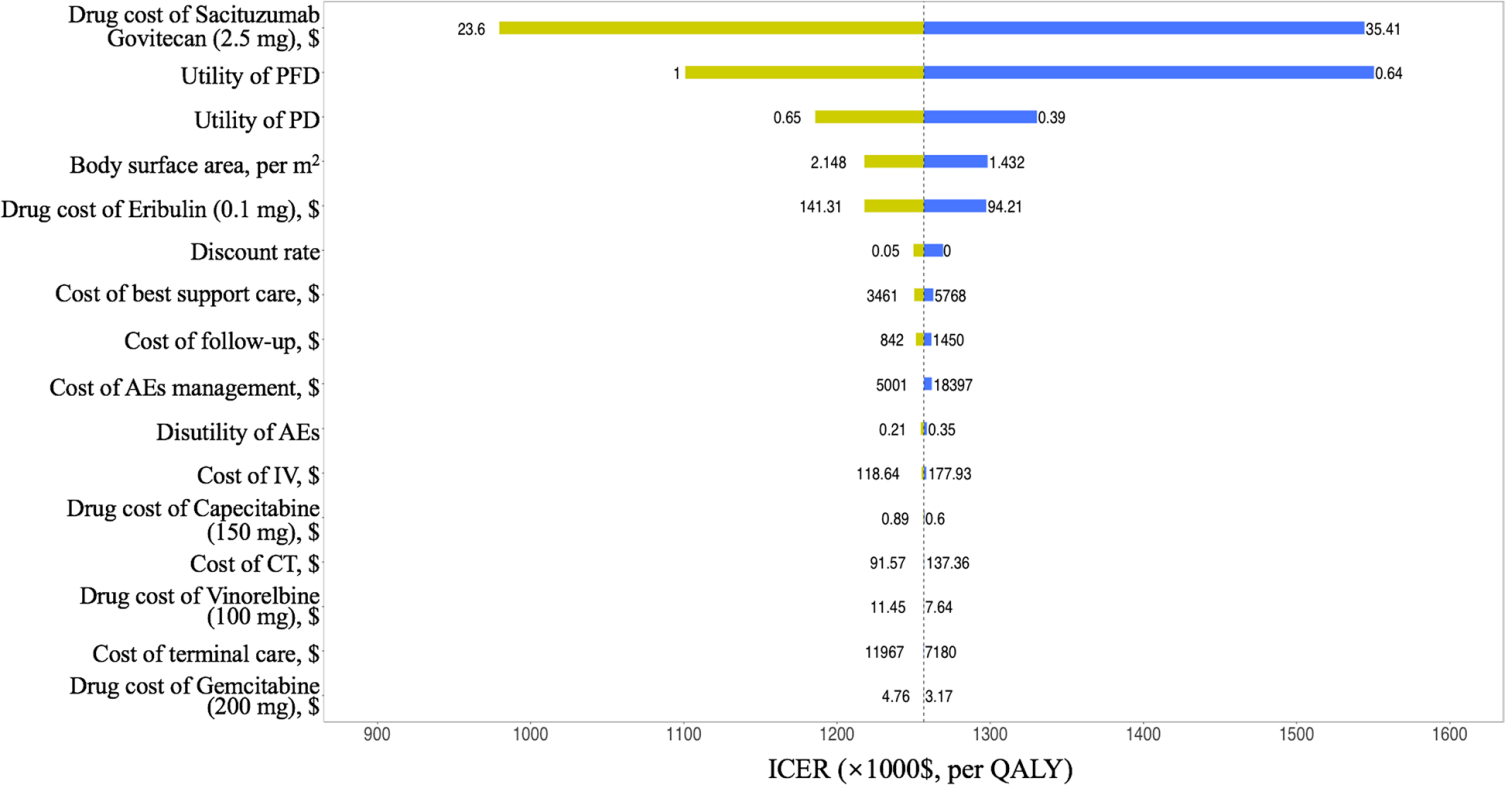
Tornado diagram for univariable sensitivity analysis. *ICER, Incremental cost-effectiveness ratio; PFD = progression-free disease; PD = progressed disease; AEs, Adverse events

**Fig. 3:**
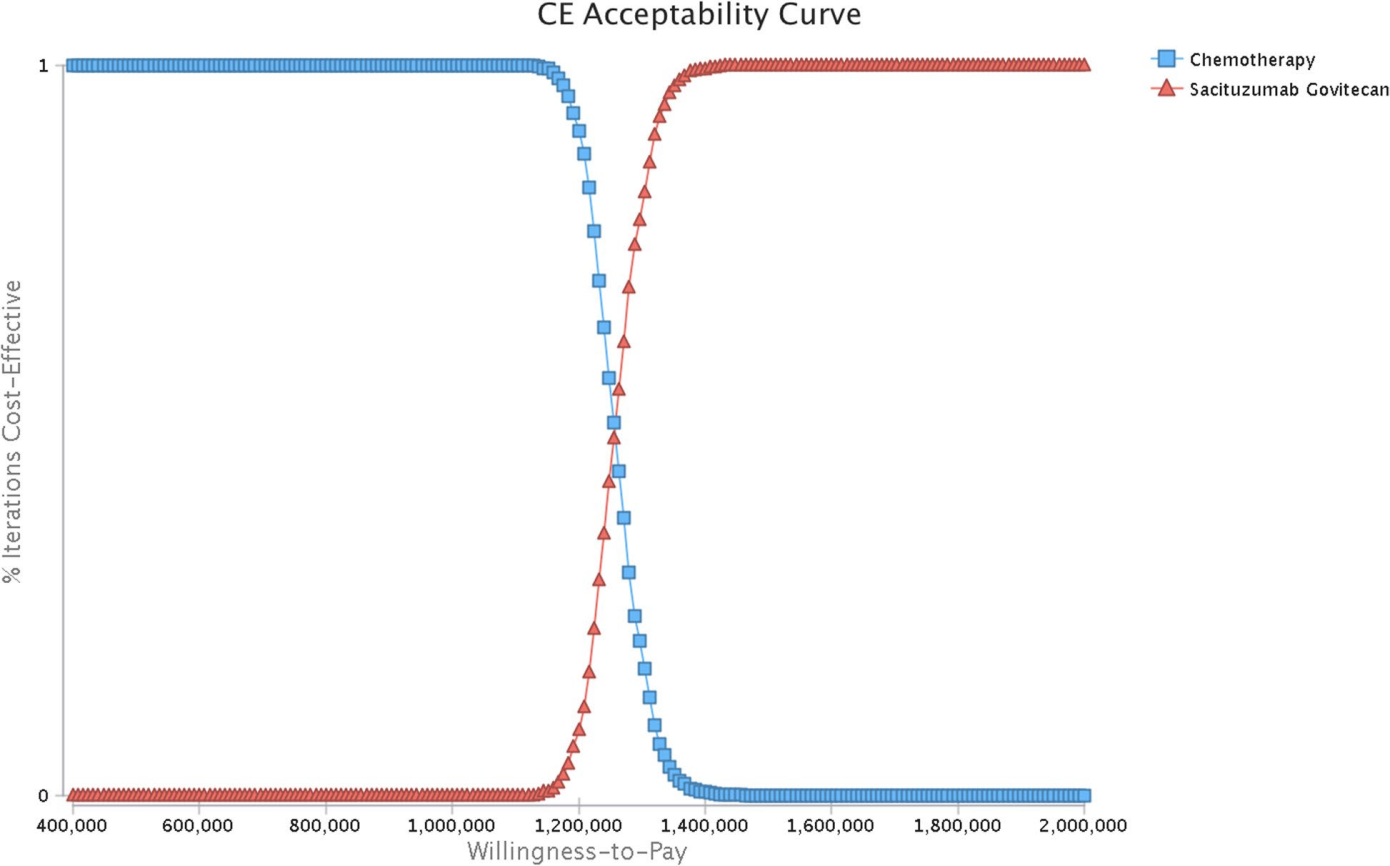
Acceptability curve of the probability sensitivity analysis among full population

The results of the univariate sensitivity analysis are displayed in Fig. 2 and demonstrate that the cost of sacituzumab govitecan, the utility of PFD, and the utility of PD were the primary drivers of the model outcomes. Other parameters, such as the body surface area of patients, utility of PD, cost of AE management, cost of BSC and drug price of chemotherapy, had moderate or mild effects on the ICER. Figure 3 shows the PSA results from the whole population. And eTable 6 in Supplementary list the mean probabilistic results for each arm and the incremental results for the comparison of sacituzumab govitecan versus chemotherapy in the full population and patients without brain metastasis, respectively. The mean probabilistic ICER of sacituzumab govitecan vs chemotherapy in the full population ($1,257,157/QALY) is slightly higer to their relevant deterministic base-case ICER ($1,252,295/QALY). The mean probabilistic ICER of pembrolizumab vs chemotherapy in the patients without brain metastasis cohort ($1,168,784/QALY) is lower than their deterministic base-case ICERs ($1,177,171/QALY). The differences in incremental LYs, and consequently incremental QALYs, are most likely caused by the uncertainty surrounding survival modeling, which lead to the differences between deterministic and probabilistic outcomes. However, sacituzumab govitecan (vs chemotherapy) is unlikelihood to be cost-effective at a WTP threshold of $150,000/QALYs. Only if the WTP threshold was increased to $1,320,000/QALYs would sacituzumab govitecan have a 90% probability of being considered a cost-effective treatment compared with chemotherapy.

### Scenario analyses

We explored younger and older baseline ages in scenario 1. As a younger age of 18 years meant a longer life expectancy with more time to accrue incremental benefits from disease progression, the ICER for sacituzumab govitecan vs chemotherapy s slightly decreased to $1,242,295/QALYs. Conversely, increasing the baseline age to 80 years allowed for less time to accrue disease benefits, increasing the ICER for sacituzumab govitecan vs chemotherapy to $1,561,370/QALYs. In scenario 2, the time horizon was varied to 1, 3, and 5 years, and the ICERs were $1,240,130/QALYs, $1,138,634/QALYs, and $1,201,472/QALYs, respectively. In scenario 3, the ICERs for sacituzumab govitecan vs chemotherapy were $1,102,381/QALYs or $866,663/QALYs when modeling only 80% or 50% of patients receiving BSC. In the final scenario, we found that reductions in the drug prices for sacituzumab govitecan of 20%, 50% and 80% would result in lower ICERs of 963,626/QALYs, $555,060/QALYs, and 137,308/QALYs, respectively. The results of the four scenario analyses are reported in eTable 4 in the Supplement.

## Discussion

The clinical benefits of sacituzumab govitecan treatment described in the ASCENT trial caused increased interest among oncologists, policy makers and patients [14]. However, the price of a novel anticancer drug should not only be reasonable and affordable for patients so that they can easily receive treatment but also be sustainable for national healthcare systems, reimbursement platforms, and pharmaceutical companies [34]. Because of the large need for treating TNBC and the rising concern over the cost-effectiveness of healthcare interventions, the unmet demand for a precise value assessment of sacituzumab govitecan use in clinical practice inspired this research.

This study compared the cost-effectiveness of sacituzumab govitecan to chemotherapy for TNBC patients with or without brain metastases. A three-state transition model was developed to simulate patients’ transition process. And the survival models were also established to perform exploratory scenario analyses for long-term outcomes. In accordance with the base-case analyses, the ICER of sacituzumab govitecan versus chemotherapy exceeds the current WTP threshold in the US ($150,000/QALY) for both cohorts (full population and patients without brain metastasis). The ICER value at baseline is lower in the cohort of patients without brain metastases compared to the total population cohort. These findings echoed a previous systematic review, which comprehensively synthesized 37 economic evaluation studies and reviewed the cost-effectiveness of 70 interventions for patients with breast cancer [35]. Evidence of heterogeneity in the cost-effectiveness of treatment and treatment selection for breast cancer was observed as a result of variability in the choice of comparators, context, whether therapy was used in the adjuvant or metastatic setting, patient population subtype, and perspective. However, nearly half of the 70 treatments evaluated across 37 therapeutic studies showed that the intervention of interest did not have acceptable costs per QALY for the country of analysis, despite the fact that the threshold for being considered cost-effective differed by setting and country [35]. Moreover, this review also reported that the ICERs in the metastatic phase of therapy were less favorable than those in the adjuvant period due to increased drug expenditures and lower QALY increases [35].

All the sensitivity analyses demonstrated the robustness of the model based on the uncertainty of the model variables. There was only a marginal difference among the baseline results from the whole population and from patients without brain metastasis. Therefore, we presented only the results of the univariate sensitivity analysis from the whole population. The univariate sensitivity analysis revealed that the price of sacituzumab govitecan was the most influential factor for the model outcomes. Therefore, we changed the price of sacituzumab govitecan in scenario analysis 4. And the results indicated that the ICER decreased to $963,626/QALY, $555,060/QALY, and $137,308/QALYs when we reduced the cost of sacituzumab govitecan to 20%, 50%, and 80% of its original price. In that scenario, sacituzumab govitecan was cost-effective at the current US WTP threshold of $150,000/QALYs compared with chemotherapy only if the cost of sacituzumab govitecan was reduced to 80% of its original price. The consistency between the mean PSA output and the base case results further demonstrates this analysis's robustness. And the PSA demonstrated that sacituzumab govitecan versus chemotherapy is unlikely to be cost-effective at a WTP threshold of $150,000/QALYs; unless the WTP threshold was raised to $1,320,000/QALYs, sacituzumab govitecan could have a 90% chance of being deemed cost-effective compared to chemotherapy. Our findings can provide important information to patients, doctors, and health care decision makers and are critical for both developed and developing countries.

The strengths of our study are worth highlighting. First, to our knowledge, this was the first study to simultaneously evaluate the health and economic outcomes of sacituzumab govitecan for metastatic TNBC by integrating the latest evidence through a decision-making model approach. Although sacituzumab govitecan is a novel agent in metastatic TNBC and its promising outcomes reported in previous studies have been confirmed, the economic value of sacituzumab govitecan in metastatic TNBC is still unknown. Second, we stratified patients according to the presence of brain metastasis in the baseline analysis and conducted a series of scenario analyses for the whole population to reflect the situation in clinical practice (i.e., simulating the circumstance that some patients will not receive BSC due to other causes). Third, our study was performed by adopting a microsimulation model to account for the heterogeneity of patients.

There are also several limitations that should be considered. First, health benefits over the observational period of the ASCENT trial were extrapolated by fitting parametric distributions to the reported PFS and OS data, which might have led to uncertainty in the model outcomes, although the observed and reconstructed data were validated. It is necessary to evaluate the concordance of these modeled health outcomes with real-world data and long-term RCTs. Second, the ASCENT trial did not report information on the quality of life (utility) of patients; therefore, the utility values were collected from a previously published cost-effectiveness analysis of TNBC, and there may be some differences from the real-world data due to different patient characteristics. Third, we assumed that patients received BSC after disease progression; however, the treatment sequence is more diversified and individualized in clinical practice. Notwithstanding these limitations, because the findings of this study reflect the general clinical practices of managing metastatic TNBC, they might be a critical and valuable reference for patients, physicians and policy makers.

## Conclusion

In summary, for relapsed or refractory metastatic TNBC patients, the second-line therapy approach of sacituzumab govitecan should not be considered a cost-effective option at the current WTP threshold of $150,000 in the US.

## Supplementary Information

**Additional file 1: eTable 1.** Baseline Patient Characteristics. **eTable 2.** Drug dose and costs. **eTable 3.** Background mortality rate. **eTable 4.** Scenario analyses in full population. **eTable 5.** The variance–covariance matrix and Cholesky equation with random normal distribution of all survival parameters. **eTable 6.** Probabilistic results. **eFigure 1.** Parametric Distributions of Overall Survival for Full Population. **eFigure 2.** Parametric Distributions of Progression-free Survival for Full Population. **eFigure 3.** Parametric Distributions of Overall Survival for Patients Without Brain metastatic. **eFigure 4.** Parametric Distributions of Progression-free Survival for Patients Without Brain metastatic.

## Data Availability

All data generated or analyzed during this study are included in this published article/as Supplementary information files.
