# Supplementary material for: Cost-effectiveness of sacituzumab govitecan versus chemotherapy in patients with relapsed or refractory metastatic triple-negative breast cancer
Source: BMC Health Serv Res. 2023 Jun 29;23:706. doi: 10.1186/s12913-023-09728-6 (PMC10308729; doi:10.1186/s12913-023-09728-6)

**eTable 1. Baseline Patient Characteristics.**

| Patient characteristic | Sacituzumab Govitecan (N = 235) | Chemontherapy (N = 235) |
| --- | --- | --- |
| Median age, yr | 54 | 53 |
| Sex, no. (%) | 233 (99) | 233 (100) |
| Race or ethnic group, no. (%) |  |  |
| White | 188 (80) | 181 (78) |
| Black | 28 (12) | 28 (12) |
| Asian | 9 (4) | 9 (4) |
| Other or not Specified | 10 (4) | 15 (6) |
| ECOG performance-status score at screening, no. (%) |  |  |
| 0 | 108 (46) | 98 (42) |
| 1 | 127 (54) | 135 (58) |
| Previous chemotherapy regimens, no. (%) |  |  |
| 2 or 3 | 166 (71) | 164 (70) |
| >3 | 69 (29) | 69 (30) |
| Previous use of PD-1 or PD-L1 inhibitors, no. (%) | 67 (29) | 60 (26) |

**eTable 2. Drug dose and costs**

| Drug | Dose | Route | Unit Price ($) | Cost for 1 model cycle ($, 21 days) |
| --- | --- | --- | --- | --- |
| sacituzumab govitecan | 10 mg/kg of body weight was administered on days 1 and 8 of a 21-day cycle | IV | 11.80/mg | 16,520 |
| Chemotherapy | | | | |
| Eribulin | 1.4 mg/m^2^ of body-surface area on days 1, and 8 of a 21-day cycle | IV | 1177.62/mg | 5,902.2 |
| vinorelbine | 25 mg/m^2^ intravenously on day 1 weekly | IV | 0.95/mg | 127.5 |
| capecitabine | 1000 to 1250 mg/m^2^ twice daily on days 1 to 14 of a 21-day cycle | Oral | 0.745/150mg | 280 |
| gemcitabine | 800 to 1200 mg/m^2^ on days 1, 8, and 15 of a 28-day cycle | IV | 3.97/200mg | 44.7 |

**eTable 3. Background mortality rate**

Estimates of background mortality rate for each age are provided in the US life table; Arias E, Heron M, Xu J. United States Life Tables, 2019. Natl Vital Stat Rep. 2019; 68:1-65.

| Age (years) | Background | Age (years) | Background | Age (years) | Background |
| --- | --- | --- | --- | --- | --- |
| 18 | 0.000603 | 54 | 0.007003 | 90 | 0.166829 |
| 19 | 0.000698 | 55 | 0.007607 | 91 | 0.185047 |
| 20 | 0.000795 | 56 | 0.008219 | 92 | 0.204441 |
| 21 | 0.000889 | 57 | 0.008857 | 93 | 0.224919 |
| 22 | 0.000970 | 58 | 0.009542 | 94 | 0.246354 |
| 23 | 0.001424 | 59 | 0.010285 | 95 | 0.26890 |
| 24 | 0.001497 | 60 | 0.011098 | 96 | 0.291442 |
| 25 | 0.001561 | 61 | 0.011952 | 97 | 0.314700 |
| 26 | 0.001624 | 62 | 0.012814 | 98 | 0.338142 |
| 27 | 0.001682 | 63 | 0.013657 | 99 | 0.361537 |
| 28 | 0.001737 | 64 | 0.014502 | 100 | 1 |
| 29 | 0.001792 | 65 | 0.015384 |  |  |
| 30 | 0.001847 | 66 | 0.016444 |  |  |
| 31 | 0.001900 | 67 | 0.017624 |  |  |
| 32 | 0.001952 | 68 | 0.018968 |  |  |
| 33 | 0.002003 | 69 | 0.029586 |  |  |
| 34 | 0.002053 | 70 | 0.022109 |  |  |
| 35 | 0.002111 | 71 | 0.024359 |  |  |
| 36 | 0.002174 | 72 | 0.026347 |  |  |
| 37 | 0.002233 | 73 | 0.028810 |  |  |
| 38 | 0.002285 | 74 | 0.031309 |  |  |
| 39 | 0.002340 | 75 | 0.034486 |  |  |
| 40 | 0.002413 | 76 | 0.038026 |  |  |
| 41 | 0.002516 | 77 | 0.042286 |  |  |
| 42 | 0.002649 | 78 | 0.046547 |  |  |
| 43 | 0.002811 | 79 | 0.051534 |  |  |
| 44 | 0.002999 | 80 | 0.057008 |  |  |
| 45 | 0.003203 | 81 | 0.062923 |  |  |
| 46 | 0.003433 | 82 | 0.069911 |  |  |
| 47 | 0.003709 | 83 | 0.078099 |  |  |
| 48 | 0.004047 | 84 | 0.086754 |  |  |
| 49 | 0.004445 | 85 | 0.096549 |  |  |
| 50 | 0.004874 | 86 | 0.106472 |  |  |
| 51 | 0.005331 | 87 | 0.119677 |  |  |
| 52 | 0.005844 | 88 | 0.134128 |  |  |
| 53 | 0.006408 | 89 | 0.149846 |  |  |

eTable 4. Scenario analyses in full population.

| Scenarios | Description | Cost | | QALY | | ICER |
| --- | --- | --- | --- | --- | --- | --- |
|  |  | Sacituzumab  Govitecan | Chemotherapy | Sacituzumab  Govitecan | Chemotherapy |  |
| 1-Baseline age | | | | | | |
| 1-1-Upper limit of baseline age  Base case = 54 | Upper limit of age = 80 | 313206 | 90083 | 0.58 | 0.44 | 1561370 |
| 1-2-Lower limit of baseline age  Base case = 54 | Lower limit of age = 18 | 395933 | 102030 | 0.73 | 0.49 | 1242295 |
| 2-The time horizon | | | | | | |
| 2-1-Time horizon=1 |  | 252807 | 68482 | 0.52 | 0.37 | 1240130 |
| Base case value=10 |  |  |  |  |  |  |
| 2-2-Time horizon=3 |  | 388153 | 93339 | 0.72 | 0.46 | 1138634 |
| Base case value=10 |  |  |  |  |  |  |
| 2-3-Time horizon=5 |  | 396396 | 98871 | 0.73 | 0.48 | 1201472 |
| Base case value=10 |  |  |  |  |  |  |
| 3-Discontinuation |  |  |  |  |  |  |
| 3-1-80% patients receive BSC |  | 343591 | 86466 | 0.73 | 0.50 | 1102381 |
| Base case percentage=100% |  |  |  |  |  |  |
| 3-2-50% patients receive BSC |  | 266728 | 62215 | 0.73 | 0.49 | 866663 |
| Base case percentage=100% |  |  |  |  |  |  |
| 4-Costs | | | | | | |
| 3-5-Cost of sacituzumab govitecan is 20% lower than base case | sacituzumab govitecan cost = (2.5mg) | 332035 | 101978 | 0.73 | 0.50 | 963626 |
| 3-6-Cost of sacituzumab govitecan is 50% lower than base case | sacituzumab govitecan cost = (2.5mg) | 231689 | 102499 | 0.73 | 0.50 | 555060 |
| 3-7-Cost of sacituzumab govitecan is 80% lower than base case | sacituzumab govitecan cost = (2.5mg) | 134557 | 102410 | 0.73 | 0.50 | 137308 |

eTable 5. The variance–covariance matrix and Cholesky equation with random normal distribution of all survival parameters.

| Variable | Baseline value | Distribution | Variance–covariance matrix | | | Cholesky decomposition matrix | Cholesky equation with the random normal distribution |
| --- | --- | --- | --- | --- | --- | --- | --- |
| Survival model of full population | | | | | | | |
| Sacituzumab Govitecan | | | | | | | |
| OS | λ=15.6779  γ=1.4281 | Weibull |  | γ | λ | $\left( \begin{matrix} a & b \\ 0 & c \end{matrix} \right)$ = $\left( \begin{matrix} 0.06483411 & -0.006739474 \\ 0 & 0.052634309 \end{matrix} \right)$ | λ=15.6779+0.06483411🞨Z_1_  γ=1.4281+(-0.006739474)🞨Z_1_+0.052634309🞨Z_2_ |
|  |  |  | γ | 0.0042034616 | -0.0004369478 |  |  |
|  |  |  | λ | -0.0004369478 | 0.002815791 |  |  |
| PFS | λ=4.675  γ=1.739 | Loglogistic |  | γ | λ | $\left( \begin{matrix} a & b \\ 0 & c \end{matrix} \right)$ = $\left( \begin{matrix} 0.0734094 & -0.01906949 \\ 0 & 0.08488012 \end{matrix} \right)$ | λ=4.675+0.0734094🞨Z_1_  γ=1.739+(-0.01906949)🞨Z_1_+0.08488012🞨Z_2_ |
|  |  |  | γ | 0.005388939 | -0.001399880 |  |  |
|  |  |  | λ | -0.001399880 | 0.007568281 |  |  |
| Chemotherapy | | | | | | | |
| OS | λ=6.636  γ=1.933 | Loglogistic |  | γ | λ | $\left( \begin{matrix} a & b \\ 0 & c \end{matrix} \right)$ = $\left( \begin{matrix} 0.05791568 & -0.002088113 \\ 0 & 0.058110414 \end{matrix} \right)$ | λ=6.636+0.05791568🞨Z_1_  γ=1.933+(-0.002088113)🞨Z_1_+0.058110414🞨Z_2_ |
|  |  |  | γ | 0.0033542263 | -0.0001209345 |  |  |
|  |  |  | λ | -0.0001209345 | 0.0033811804 |  |  |
| PFS | λ=2.348  γ=2.251 | Loglogistic |  | γ | λ | $\left( \begin{matrix} a & b \\ 0 & c \end{matrix} \right)$ = $\left( \begin{matrix} 0.09821356 & -0.0495592 \\ 0 & 0.09054755 \end{matrix} \right)$ | λ=2.348+0.09821356🞨Z_1_  γ=2.251+(-0.0495592)🞨Z_1_+0.09054755🞨Z_2_ |
|  |  |  | γ | 0.009645904 | -0.004867385 |  |  |
|  |  |  | λ | -0.004867385 | 0.010654972 |  |  |
| Survival model of no brain metastases | | | | | | | |
| Sacituzumab Govitecan | | | | | | | |
| OS | λ=16.598  γ=1.445 | Weibull |  | γ | λ | $\left( \begin{matrix} a & b \\ 0 & c \end{matrix} \right)$ = $\left( \begin{matrix} 0.08194637 & -0.02551179 \\ 0 & c \end{matrix} \right)$ | λ=16.598+0.08194637🞨Z_1_  γ=1.445+(-0.02551179)🞨Z_1_+0.02551179🞨Z_2_ |
|  |  |  | γ | 0.006715208 | -0.002090599 |  |  |
|  |  |  | λ | -0.002090599 | 0.005211876 |  |  |
| PFS | λ=5.152  γ=1.799 | Loglogistic |  | γ | λ | $\left( \begin{matrix} a & b \\ 0 & c \end{matrix} \right)$ = $\left( \begin{matrix} 0.07258686 & -0.01185061 \\ 0 & 0.08123746 \end{matrix} \right)$ | λ=5.152+0.07258686🞨Z_1_  γ=1.799+(-0.01185061)🞨Z_1_+0.08123746🞨Z_2_ |
|  |  |  | γ | 0.0052688525 | -0.0008601989 |  |  |
|  |  |  | λ | -0.0008601989 | 0.0067399620 |  |  |
| Chemotherapy | | | | | | | |
| OS | λ=6.759  γ=1.87 | Loglogistic |  | γ | λ | $\left( \begin{matrix} a & b \\ 0 & c \end{matrix} \right)$ = $\left( \begin{matrix} 0.07451351 & -0.01310083 \\ 0 & 0.07575577 \end{matrix} \right)$ | λ=6.759+0.07451351🞨Z_1_  γ=1.87+(-0.01310083)🞨Z_1_+0.07575577🞨Z_2_ |
|  |  |  | γ | 0.0055522638 | -0.0009761885 |  |  |
|  |  |  | λ | -0.0009761885 | 0.0059105679 |  |  |
| PFS | λ=2.266  γ=2.552 | Loglogistic |  | γ | λ | $\left( \begin{matrix} a & b \\ 0 & c \end{matrix} \right)$ = $\left( \begin{matrix} 0.1008313 & -0.03686553 \\ 0 & 0.08715425 \end{matrix} \right)$ | λ=2.266+0.1008313🞨Z_1_  γ=2.552+(-0.03686553)🞨Z_1_+0.08715425🞨Z_2_ |
|  |  |  | γ | 0.010166942 | -0.003717198 |  |  |
|  |  |  | λ | -0.003717198 | 0.008954931 |  |  |
| OS = Overall survival; PFS = Progression-free survival; γ = shape parameter; λ = scale parameter; Z_1,_Z_2_ =random variables with normal distribution. | | | | | | | |

eTable 6. Probabilistic results

| Arm | Total LYs | Total QALYs | Total Costs | Inc. LYs | Inc. QALYs | Inc. Costs | ICER/LY | ICER/QALY |
| --- | --- | --- | --- | --- | --- | --- | --- | --- |
| Full population (SD; 95% CI) | | | | | | | |  |
| Chemotherapy | 0.8202 (0.02147; 95% CI [0.8192, 0.8417]) | 0.4969 (0.01188; 95% CI [0.4962, 0.4977]) | 102,442 (2877; 95% CI [102264, 102620]) | - | - | - | - | - |
| Sacituzumab govitecan | 1.1374 (0.01744; 95% CI [1.1366, 1.1548]) | 0.7297 (0.4776; 95% CI [0.7255, 0.7339]) | 395,470 (294,420; 95% CI [392889, 398051]) | 0.3172 (0.02119; 95%CI [0.3163, 0.3384]) | 0.2339 (0.01155; 95% CI [0.2332, 0.2346]) | 294,049 (6108; 95% CI [293670, 294427]) | 927,015 | 1,257,157 |
| Patients without BM (SD; 95% CI) | | | | | | | |  |
| Chemotherapy | 0.8546 (0.03230; 95% CI [0.8526, 0.8566]) | 0.5146 (0.5762, 95% CI [0.5095, 0.5196]) | 107,418 (4331; 95% CI [107151, 107687]) | - | - | - | - | - |
| Sacituzumab govitecan | 1.2010 (0.02598; 95% CI [1.1994, 1.2027]) | 0.7750 (0.01622, 95% CI [0.7739, 0.7859]) | 417,030 (10,072; 95% CI [416406, 417654]) | 0.3465 (0.03269; 95% CI [0.3445, 0.3485]) | 0.2649 (0.01733; 95% CI [0.2638, 0.2660]) | 309,611 (9302; 95% CI [309034, 310188]) | 893,538 | 1,168,784 |

eFigure 1: Parametric Distributions of Overall Survival for Full Population.


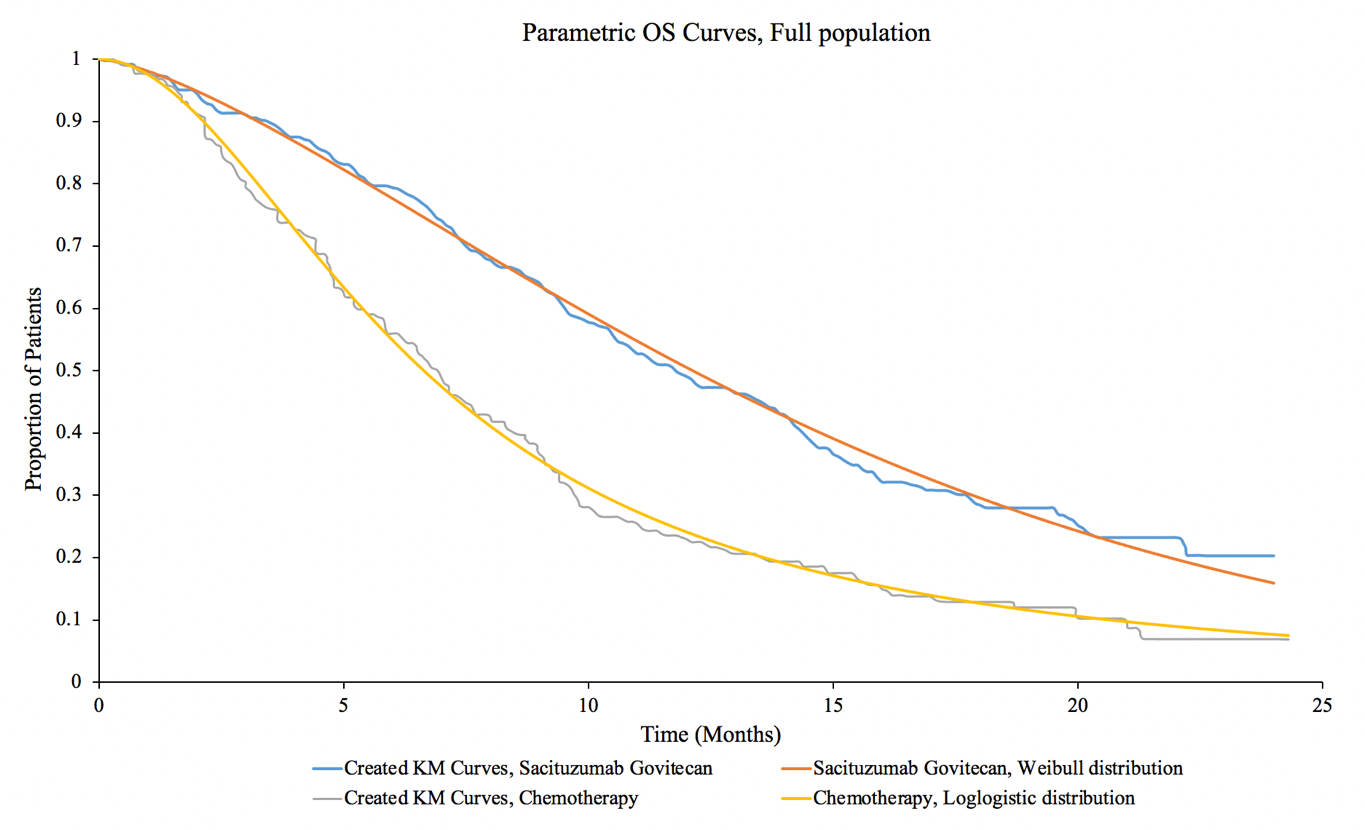


eFigure 2: Parametric Distributions of Progression-free Survival for Full Population.


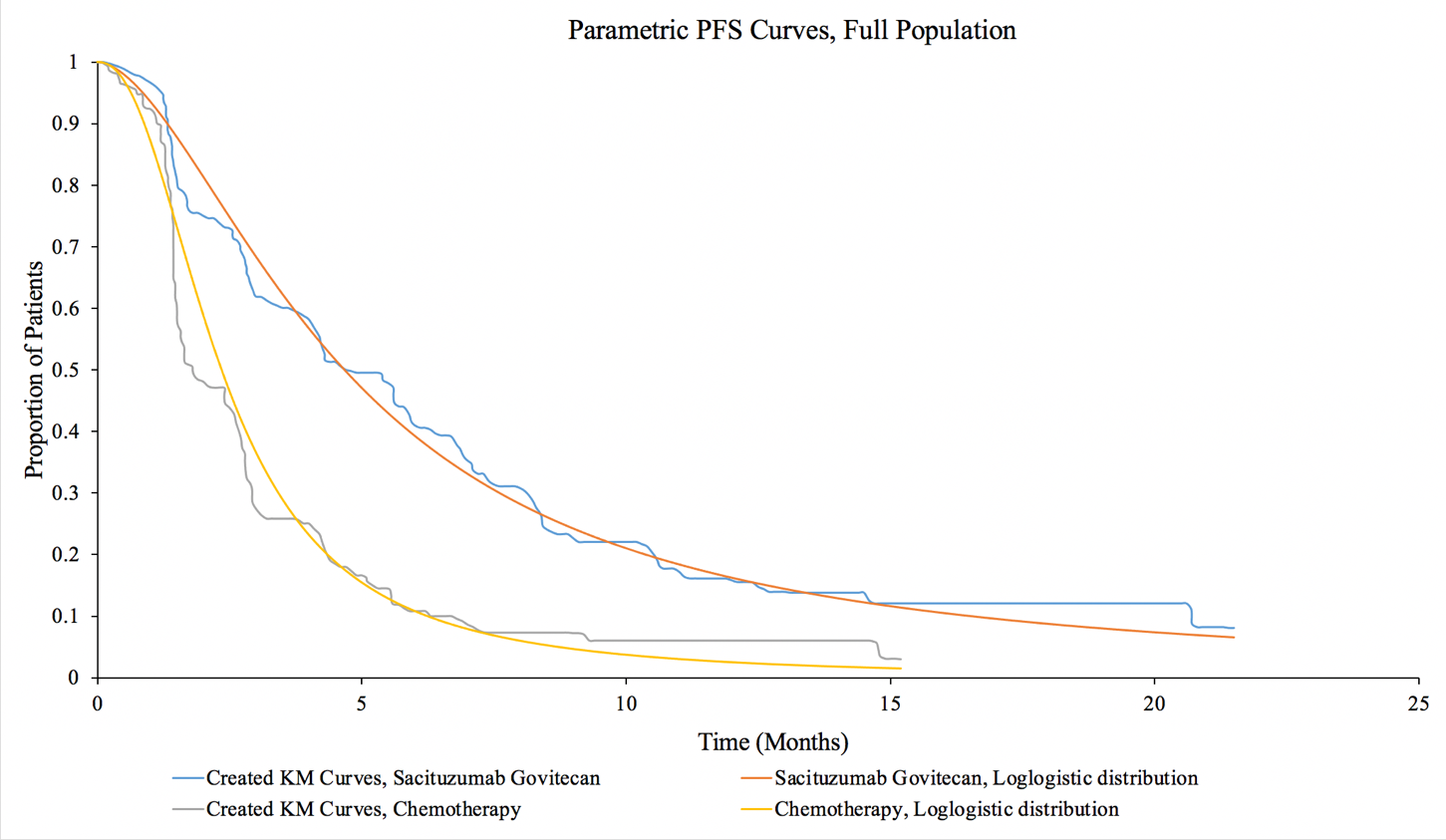


eFigure 3: Parametric Distributions of Overall Survival for Patients Without Brain metastatic.


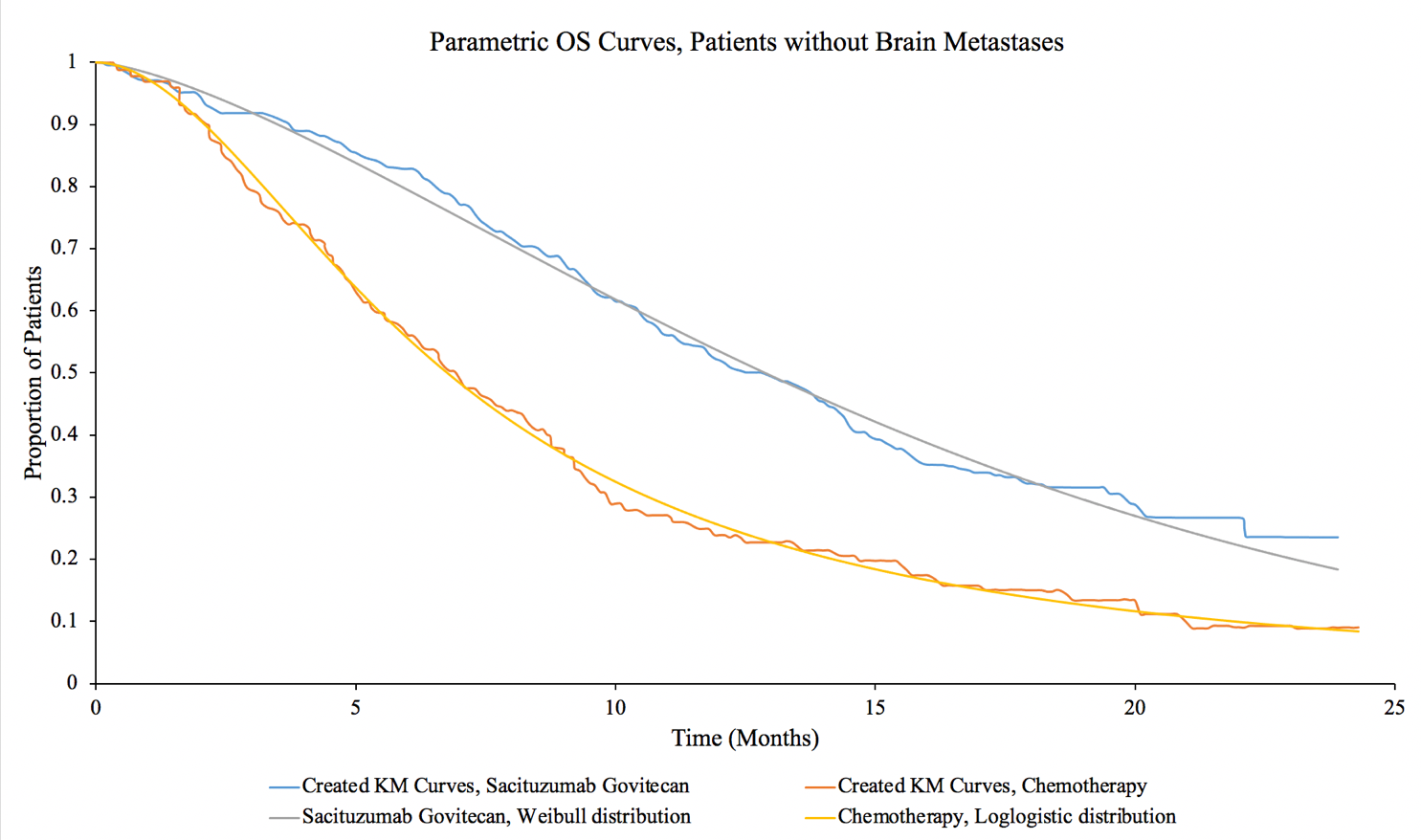


eFigure 4: Parametric Distributions of Progression-free Survival for Patients Without Brain metastatic.


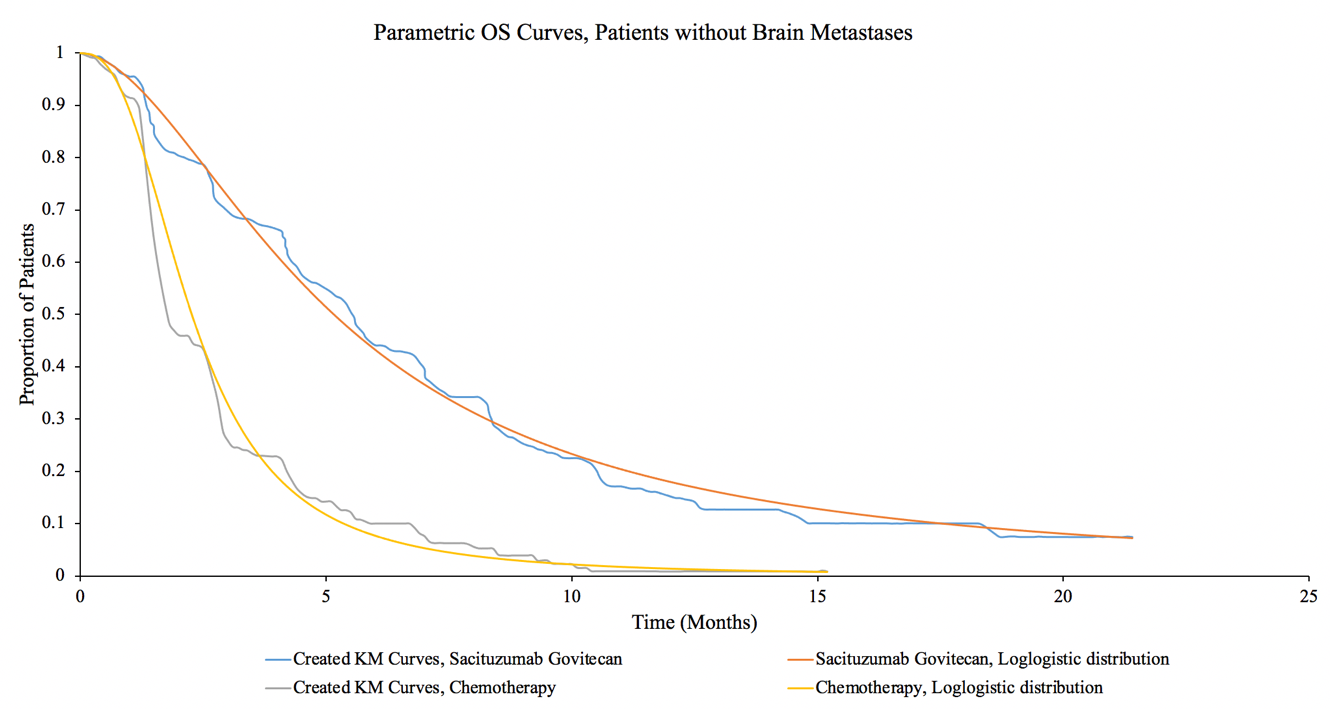

Supplement: Supplementary file 1 — Additional file 1: eTable 1. Baseline Patient Characteristics. eTable 2. Drug dose and costs. eTable 3. Background mortality rate. eTable 4. Scenario analyses in full population. eTable 5. The variance–covariance matrix and Cholesky equation with random normal distribution of all survival parameters. eTable 6. Probabilistic results. eFigure 1. Parametric Distributions of Overall Survival for Full Population. eFigure 2. Parametric Distributions of Progression-free Survival for Full Population. eFigure 3. Parametric Distributions of Overall Survival for Patients Without Brain metastatic. eFigure 4. Parametric Distributions of Progression-free Survival for Patients Without Brain metastatic. [file 12913_2023_9728_MOESM1_ESM.docx]
